# Same-Day Associations Between Perceived Stress and Suicidal Ideation Among College Students: The Roles of Social Connectedness and Self-Disgust in a 7-Day Diary Study

**DOI:** 10.3390/bs16081284

**Published:** 2026-07-28

**Authors:** Zhongjie Wang, Xinyang Xu, Qiuli Pan, Yajuan Li, Liya Huang

**Affiliations:** School of Education, Zhengzhou University, Zhengzhou 450001, China; zhongjie1597@zzu.edu.cn (Z.W.); p15836151721@gs.zzu.edu.cn (Q.P.); ly6637@gs.zzu.edu.cn (Y.L.); huangliya@gs.zzu.edu.cn (L.H.)

**Keywords:** suicidal ideation, perceived stress, social connectedness, self-disgust, serial indirect association, daily diary study, college students

## Abstract

Suicidal ideation may fluctuate over short periods, yet cross-sectional or long-interval study designs offer limited insight into day-level variation and same-day associations. This 7-day diary study examined same-day associations among perceived stress, social connectedness, self-disgust, and suicidal ideation in college students, with a particular focus on indirect association patterns involving self-disgust and social connectedness. Sixty-eight Chinese college students were recruited online; 61 provided valid data, yielding 427 daily observations. Multilevel structural equation modeling showed that, at the within-person level, daily perceived stress was associated with higher same-day self-disgust (B = 0.574, SE = 0.065, *p* < 0.001), which was associated with higher same-day suicidal ideation (B = 0.403, SE = 0.089, *p* < 0.001). The indirect association through self-disgust was significant (B = 0.232, 95% CI [0.123, 0.341]). Daily perceived stress was also associated with lower same-day social connectedness (B = −0.471, SE = 0.044, *p* < 0.001), and daily social connectedness was negatively associated with same-day self-disgust (B = −0.503, SE = 0.045, *p* < 0.001). The serial indirect association was significant (B = 0.081, 95% CI [0.043, 0.118]). These findings highlight a within-person association pattern in which higher daily perceived stress was associated with same-day suicidal ideation in relation to lower social connectedness and higher self-disgust. By contrast, the corresponding indirect associations at the between-person and trait levels were not statistically significant, suggesting that the findings should be interpreted primarily as within-person patterns of same-day associations.

## 1. Introduction

Recent evidence indicates that suicidal ideation remains a significant public health concern among Chinese university students, with the 12-month prevalence ranging from 3.89% to 5.81% between 2021 and 2023 (Yao et al., 2024). Suicidal ideation is a key cognitive manifestation within the suicidality spectrum, referring to thoughts, considerations, or plans about ending one’s life (Jobes et al., 2024). As an important subjective signal in the development of suicide risk, suicidal ideation not only varies between individuals but may also fluctuate over short periods in daily life alongside changes in stress and affective–cognitive states (Kleiman et al., 2017). Most existing studies have relied on cross-sectional designs or longitudinal designs with relatively long measurement intervals. Although these studies have revealed general associations among stress, negative self-evaluation, and suicidal ideation, they are less able to address a more day-level question: whether an individual’s suicidal ideation increases on days when their perceived stress is higher than usual (Love & Morgan, 2024).

In recent years, daily diary methods have been increasingly applied in suicide research to capture day-level variations in individuals’ experiences in naturalistic contexts (Kivelä et al., 2022; Czyz et al., 2023). Unlike EMA designs that typically involve multiple assessments per day and are particularly suited to examining rapid within-day fluctuations, once-daily diary designs are well suited for examining day-level variation and same-day associations (Bolger & Laurenceau, 2013; Conner & Barrett, 2012). Further evidence suggests that incorporating time-varying information, such as daily affective fluctuations and stressful events, may improve the prediction of subsequent suicidal ideation among young people (Lei et al., 2023). Theoretically, the integrated motivational–volitional model emphasizes that stressful experiences may contribute to suicidal ideation through self-related appraisals involving defeat, humiliation, and entrapment, whereas the three-step theory highlights the roles of psychological pain and connectedness in the emergence of suicidal ideation (Klonsky et al., 2021; O’Connor & Kirtley, 2018). Although intensive longitudinal studies have documented substantial short-term variability in suicidal ideation and its affective and interpersonal correlates (Kleiman et al., 2017; Kivelä et al., 2022; Winstone et al., 2024), comparatively little work has examined daily perceived stress, social connectedness, and self-disgust together within the same study. To address this gap, the present study used a 7-day daily diary design to examine the same-day associations among daily perceived stress, social connectedness, self-disgust, and suicidal ideation.

Perceived stress refers to an individual’s subjective appraisal of the extent to which life circumstances are unpredictable, uncontrollable, and exceed their available coping resources (Cohen et al., 1983; Lazarus & Folkman, 1984). From the perspective of cognitive appraisal theory, the psychological impact of stress depends not only on the objective event itself, but also on whether individuals appraise the situation as exceeding their capacity to cope (Lazarus & Folkman, 1984). Recent longitudinal studies among college students have also shown that stress experiences may influence subsequent psychological adjustment through cognitive appraisal processes, further supporting the subjective appraisal nature of perceived stress (Simães et al., 2024; Spătaru et al., 2024). Thus, perceived stress may more directly reflect the psychological burden that individuals experience in daily life. A cross-lagged study of Chinese college students found a significant longitudinal association between perceived stress and subsequent suicidal ideation, suggesting that higher perceived stress may be an important risk factor for the development of suicidal ideation in this population (Xu et al., 2022). In addition, another study among Chinese college students showed that interpersonal stress predicted suicidal ideation by undermining meaning in life, indicating that stress experiences and their subjective meaning-making processes may constitute important sources of risk for suicidal ideation among college students (Yu & He, 2023). However, existing studies have primarily relied on cross-sectional or traditional longitudinal designs, which reveal overall associations between perceived stress and suicidal ideation but are less able to capture whether short-term increases in stress are accompanied by changes in suicidal ideation within the same individual in daily life. Accordingly, the present study further examined the association between daily perceived stress and same-day suicidal ideation from a within-person day-level perspective.

Stress experiences do not necessarily translate directly into suicidal ideation; rather, their effects may depend on how individuals interpret stressful situations and their implications for the self (O’Connor & Kirtley, 2018). When stress is interpreted as evidence of personal inadequacy, failure, or unacceptability, individuals may be more likely to experience self-directed disgust and rejection, which may be closely related to suicidal ideation (O’Connor & Kirtley, 2018; Souza et al., 2024). Self-disgust is a self-directed negative self-conscious emotion, characterized by disgust, rejection, and devaluation toward one’s physical self, overall self, or behavior (Overton et al., 2008; Ypsilanti et al., 2019). Compared with general negative affect, self-disgust more directly involves self-evaluative appraisals of self-worth, self-acceptance, and the perceived acceptability of the self, and may therefore represent a specific self-evaluative correlate of stress-related suicidal ideation (Gao et al., 2022). Although self-disgust has often been examined as a relatively stable individual difference, recent work has also distinguished state self-disgust from trait self-disgust, suggesting that self-disgust may arise in response to specific situations or stimuli (Aristotelidou et al., 2023). This distinction is particularly relevant in a daily diary context, as daily stressful experiences may be accompanied by temporary increases in self-rejection or perceived unacceptability, especially when these experiences are appraised as reflecting personal inadequacy or failure (O’Connor & Kirtley, 2018; Souza et al., 2024). Existing research has shown moderate to strong associations between self-disgust and psychological distress, including depression and anxiety, suggesting its important role in negative affect and psychological risk (Gao et al., 2022). In suicide research, Akram et al. (2022) found that self-disgust was significantly associated with suicidal thoughts and behaviors and mediated the association between body image disturbance and suicidal thoughts and behaviors. A recent longitudinal study among Chinese adolescents further showed that emotional abuse indirectly predicted suicidal ideation through self-disgust, suggesting that self-disgust may serve as an important bridge between stressful experiences and suicidal ideation (Gong & Zhou, 2025). However, these studies have primarily focused on relatively stable individual differences or stressful experiences over longer time spans, leaving it unclear whether higher-than-usual daily stress is accompanied by higher same-day self-disgust and suicidal ideation. Accordingly, the present study examined the same-day indirect association between daily perceived stress and suicidal ideation involving daily self-disgust.

Social connectedness generally refers to individuals’ experiences of belonging, closeness, and acceptance within social relationships, and represents an important psychological indicator of relational resources (Lee et al., 2001). Prior research has shown that higher social connectedness is associated with lower psychological distress and better psychological adjustment, whereas insufficient social connectedness may be associated with loneliness, helplessness, and suicidal ideation (Ypsilanti et al., 2019). From the perspective of interpersonal theories of suicidal ideation, social connectedness is closely related to belongingness, and thwarted belongingness and interpersonal disconnection are considered important psychological foundations of suicidal ideation (Joiner, 2005; Van Orden et al., 2010). Therefore, lower social connectedness may make it more difficult for individuals to obtain emotional support, affirmation of self-worth, and relational security under stressful circumstances, thereby being associated with more negative self-evaluation, self-disgust, and suicidal ideation (Ypsilanti et al., 2019; Howlader et al., 2024). In contrast, higher social connectedness may reduce the likelihood that individuals experience self-denial and self-rejection in response to daily stress by providing a sense of acceptance, belonging, and affirmation of self-worth (Baumeister & Leary, 1995; Arango et al., 2024). At the same time, social connectedness, self-disgust, and suicidal ideation may be closely intertwined in daily life. For instance, individuals who experience stronger self-disgust may feel less acceptable to others and perceive lower relational closeness, whereas suicidal ideation may also coincide with more negative perceptions of interpersonal connection (Joiner, 2005; Van Orden et al., 2010; Ypsilanti et al., 2019). Taken together, prior theory and evidence provide a rationale for specifying the proposed sequence as a theory-guided same-day association model, while acknowledging that other orderings among these constructs are also possible. Accordingly, the present study examined whether daily social connectedness and self-disgust were involved in a same-day serial indirect association between daily perceived stress and suicidal ideation.

Based on the theoretical and empirical evidence reviewed above, the present study used a 7-day once-daily diary design to examine same-day associations among daily perceived stress, social connectedness, self-disgust, and suicidal ideation among college students. Daily diary designs repeatedly assess individuals’ emotional, cognitive, and behavioral states in naturalistic contexts, making them well suited for capturing day-level variation in suicidal ideation and related psychological factors (Kivelä et al., 2022; Czyz et al., 2023; Winstone et al., 2024). Unlike traditional cross-sectional studies, the present study focused on daily within-person variation; that is, whether individuals reported lower social connectedness, higher self-disgust, and higher suicidal ideation on days when their perceived stress was higher than their usual level. Specifically, this study examined whether: (1) daily perceived stress was positively associated with same-day suicidal ideation; (2) daily self-disgust was involved in the same-day indirect association between daily perceived stress and suicidal ideation; and (3) daily social connectedness and daily self-disgust were sequentially involved in the same-day indirect association between daily perceived stress and suicidal ideation. Through this design, the present study aimed to examine hypothesized same-day relational and self-evaluative association patterns linking daily stress with suicidal ideation, and to further understand the roles of social connectedness and self-disgust in day-level variations in suicidal ideation.

## 2. Materials and Methods

### 2.1. Participants

The present study used a 7-day once-daily diary design. Participants were recruited online through convenience sampling via WeChat Moments and WeChat groups. Interested participants accessed the questionnaire through Wenjuanxing and completed an informed consent form before the baseline survey. A total of 68 Chinese college students were recruited. Participants received RMB 5 as compensation after completing the diary assessments. After invalid responses were excluded, 61 participants were retained for the final analyses, yielding an effective inclusion rate of 89.7%.

The inclusion criteria were as follows: (1) being a college student in China; (2) being able to read and complete online questionnaires independently; (3) being willing to participate in the 7-day diary study; and (4) providing informed consent. Invalid data were excluded according to the following criteria: failure to complete all daily diary assessments, unmatched participant identification numbers, response times that were clearly below a reasonable range and patterned responses. Because all questionnaire items were required, no item-level missing data occurred in the submitted questionnaires. No formal a priori power analysis was conducted; recruitment was based on the feasibility of repeated daily assessment. Given the final sample of 61 participants and 427 observations, models were restricted to fixed slopes, and the between-person and 2-1-1 findings were interpreted cautiously.

### 2.2. Procedure

The study consisted of one baseline assessment and seven consecutive daily diary assessments. Before the daily diary period began, each participant was assigned a unique study ID that did not contain personally identifying information. Participants were instructed to enter the same study ID each time they completed a questionnaire so that their baseline and daily diary responses could be matched across assessments. During the diary period, the researcher sent the daily questionnaire at approximately 19:00 each evening and reminded participants who had not yet completed the questionnaire at 22:00. Before participation, participants were informed through the informed consent form that their responses would be collected and processed in a coded/pseudonymized manner and would be reported only in aggregate form. The researchers also provided detailed instructions regarding the daily assessment schedule, response requirements, data confidentiality, and procedures for withdrawal.

### 2.3. Measures

Given the repeated nature of daily diary assessments and the need to minimize participant burden, the measures used in this study were shortened and adapted to the day level while seeking to retain coverage of the target constructs. Specifically, items were selected from established scales based on their conceptual relevance to the target constructs and their suitability for repeated daily assessment. No EFA or CFA was conducted for item selection in the present sample. Where necessary, item wording and time frames were adapted to refer to “today” so as to assess participants’ psychological states on each diary day. Higher scores on each measure indicated higher levels of the corresponding variable. Because several scales were shortened and adapted for daily assessment, the internal consistency reliability of each multi-item brief measure was re-examined and reported in the present sample. The exact item wording, response options, scoring directions, and possible score ranges are provided in Supplementary Table S1.

#### 2.3.1. Daily Suicidal Ideation

Daily suicidal ideation was assessed using an adapted version of Item 2 of the Suicidal Behaviors Questionnaire-Revised (SBQ-R; Osman et al., 2001). To correspond with the daily diary design, the original 12-month time frame was replaced with “today.” The adapted item was: “Today, how many times have you considered ending your life?” Responses were rated on a five-category frequency scale: 1 = never, 2 = rarely (once), 3 = sometimes (twice), 4 = often (three to four times), and 5 = very often (five or more times). The possible score range was 1–5, with higher scores indicating more frequent same-day suicidal ideation. Because suicidal ideation was assessed using a single item, internal consistency was not applicable and therefore was not estimated. The single-item format was used to reduce participant burden during repeated daily assessment (Eisele et al., 2022); however, its day-level reliability and validity were not independently established in the present study.

#### 2.3.2. Daily Self-Disgust

Daily self-disgust was assessed using the Chinese version of the Self-Disgust Scale, originally developed by Overton et al. and revised by Xiong et al. (Overton et al., 2008; Xiong et al., 2019). The original scale contains 12 items. To reduce participant burden during repeated daily assessment, three items were selected a priori from the Chinese version of the scale based on their content relevance to daily self-directed rejection and negative self-evaluation and their suitability for repeated assessment. The item wording was adapted to reflect participants’ state on that day. Example items included “Today, I hated being myself” and “Today, I liked the way I looked.” Items were recoded where necessary before scoring. Items were rated on a 7-point Likert scale and summed to obtain a total score, with higher scores indicating higher levels of same-day self-disgust. In the present study, the brief daily self-disgust measure showed good internal consistency across the 7-day diary assessments, with an average Cronbach’s α of 0.910, ranging from 0.861 to 0.943.

#### 2.3.3. Daily Perceived Stress

Daily perceived stress was assessed using the 10-item Perceived Stress Scale (PSS-10; Cohen et al., 1983; Lu et al., 2017). The original scale contains 10 items. To reduce participant burden during repeated daily assessment, four items were selected a priori from the PSS-10 based on their content relevance to daily experiences of unpredictability, lack of control, and coping difficulty and their suitability for repeated assessment. The item time frame was adapted to refer to “today”. Example items included “Today, I was upset because of something that happened unexpectedly” and “Today, I felt that difficulties were piling up so high that I could not overcome them.” Positively worded items were reverse-coded before scoring. Items were rated on a 5-point Likert scale and summed to obtain a total score, with higher scores indicating higher levels of same-day perceived stress. In the present study, the brief daily perceived stress measure showed good internal consistency across the 7-day diary assessments, with an average Cronbach’s α of 0.870, ranging from 0.837 to 0.908.

#### 2.3.4. Daily Social Connectedness

Daily social connectedness was assessed using the Chinese version of the Social Connectedness Scale-Revised (SCS-R), originally developed by Lee et al. and revised by Fan et al. (Lee et al., 2001; Fan et al., 2015). The original scale contains 20 items. To reduce participant burden during repeated daily assessment, four items were selected a priori from the Chinese version of the scale based on their content relevance to daily experiences of belonging, closeness, and interpersonal understanding and their suitability for repeated assessment. The item wording was adapted to reflect participants’ state on that day. Example items included “Today, I felt close to people around me” and “Today, I felt understood by people I knew.” Negatively worded items were reverse-coded before scoring. Items were rated on a 6-point Likert scale and summed to obtain a total score, with higher scores indicating higher levels of same-day social connectedness. In the present study, the brief daily social connectedness measure showed good internal consistency across the 7-day diary assessments, with an average Cronbach’s α of 0.919, ranging from 0.893 to 0.939.

### 2.4. Data Processing and Statistical Analysis

First, SPSS 27.0 was used for preliminary data screening and management, including participant identification matching, invalid response screening, missing data inspection, variable scoring, and merging of baseline and diary data. The 7-day diary data were then organized into a long-format dataset, with one observation per participant per day, to create a data structure suitable for multilevel analyses. Formal statistical analyses were conducted using R 4.5.0.

Before testing the main models, descriptive statistics were calculated, and correlations were examined at the between-person level using baseline trait scores and participants’ mean daily scores across the 7-day diary period. These correlations were reported for descriptive purposes only. Next, unconditional random-intercept models were estimated to calculate intraclass correlation coefficients (ICCs) for the day-level variables, including daily perceived stress, daily social connectedness, daily self-disgust, and daily suicidal ideation. The ICCs were used to evaluate the proportions of variance attributable to within-person and between-person levels. Because the diary data had a nested structure, with daily observations nested within individuals, multilevel models were used to test the main hypotheses.

Multilevel structural equation modeling (MSEM) was used to examine same-day associations among daily perceived stress, daily social connectedness, daily self-disgust, and daily suicidal ideation. Model analyses were conducted using the lavaan package in R (Rosseel, 2012), with the nested data structure specified using the participant identification variable. Models were estimated using robust maximum likelihood estimation (MLR). MLR provides sandwich-based robust standard errors and scaled test statistics, thereby reducing the influence of departures from multivariate normality. The final analytic dataset contained no missing values; therefore, no additional missing-data estimation procedure was required. The daily variables were not manually centered; instead, the two-level MSEM in lavaan decomposed their total variance into within-person and between-person components, thereby enabling the simultaneous estimation of within-person same-day associations and between-person differences (Curran & Bauer, 2011; Preacher et al., 2010). Trait-level predictors were standardized and entered at Level 2. Specifically, within-person paths reflect whether deviations from an individual’s usual level on a given day are associated with deviations in other variables on that same day, whereas between-person paths reflect associations among individuals’ average levels across the diary period. All within-person and between-person paths were specified as fixed effects. Random slopes were not estimated because of the modest number of Level-2 units and the increased model complexity that random-slope estimation would require.

For the mediation analysis, we first examined the multilevel indirect association involving daily perceived stress, daily self-disgust, and same-day suicidal ideation, with a focus on whether daily perceived stress was associated with same-day suicidal ideation through daily self-disgust. For the serial mediation analysis, we further tested the multilevel serial indirect association from daily perceived stress through daily social connectedness and daily self-disgust to same-day suicidal ideation. Specifically, this model tested whether, on days when individuals reported higher perceived stress than their usual level, they also reported lower social connectedness, higher self-disgust, and higher same-day suicidal ideation. Corresponding between-person paths were also estimated to examine whether individuals with higher average perceived stress differed in their average levels of social connectedness, self-disgust, and suicidal ideation.

Indirect associations were estimated as the products of path coefficients. Their 95% Wald confidence intervals were calculated using the robust standard errors obtained from the MLR estimator. An indirect association was considered statistically significant when the confidence interval did not include zero. The estimated models were just-identified (df = 0); therefore, global fit indices such as the CFI, TLI, RMSEA, and SRMR were not informative and were not interpreted. All statistical tests were two-tailed, with the significance level set at *p* < 0.05.

Age, gender, educational level, and only-child status were not included in the primary models because the hypotheses focused primarily on within-person same-day associations and the number of Level-2 units was modest. Sensitivity analyses were subsequently conducted by simultaneously including these variables as Level-2 covariates predicting social connectedness, self-disgust, and suicidal ideation. Age was grand-mean centered, whereas gender, educational level, and only-child status were dummy coded.

### 2.5. Ethical Considerations

This study was approved by the Ethics Review Committee of Zhengzhou University (Reference Number: ZZUIRB2024-021). The approved protocol covered the baseline survey, the 7-day online diary assessments, the repeated assessment of suicidal ideation, and participant support procedures. Before participation, all participants signed a written informed consent form describing the study purpose, response requirements, potential discomfort, confidentiality procedures, voluntary participation, and the right to withdraw at any time. Each participant was assigned a unique study ID at the beginning of the study, which was used to match baseline and daily diary responses. Research data were stored and analyzed in a pseudonymized format, and contact information used for questionnaire distribution, reminders, and compensation was kept separate from the analytic dataset.

Given the repeated assessment of suicidal ideation, daily responses were reviewed by the research team after submission. Responses indicating a moderate-or-higher level of suicidal ideation prompted a predefined safety response procedure, which involved contacting the participant, providing psychological support resources, and encouraging professional help-seeking when needed. This process was supervised by a psychology professor with professional counseling qualifications. Participants were not required to provide emergency contact information. Psychological support resources, including university counseling services and publicly available psychological assistance resources, were provided in the informed consent form.

## 3. Results

### 3.1. Preliminary Analyses and Correlations

The mean age of participants was 21.82 years (SD = 2.41). In terms of educational level, 73.8% were undergraduate students and 26.2% were graduate students or above. Regarding only-child status, 32.8% were only children and 67.2% were not only children.

Descriptive statistics for all key study variables are presented in Table 1. Trait perceived stress and trait social connectedness had mean scores of 31.25 (SD = 8.47; range = 12–47) and 73.67 (SD = 22.95; range = 20–111), respectively. Across the 427 daily observations, the mean scores were 10.85 (SD = 4.11) for perceived stress, 16.77 (SD = 5.18) for social connectedness, 9.62 (SD = 4.89) for self-disgust, and 1.47 (SD = 0.85) for suicidal ideation. Daily suicidal ideation was endorsed by 33 participants (54.1%) on at least one diary day and in 120 of the 427 diary entries (28.1%). Its distribution was positively skewed (skewness = 1.86).

Before testing the multilevel mediation and serial mediation models, intraclass correlation coefficients (ICCs) were first calculated for the day-level variables to examine the extent of variance at the within-person and between-person levels. As shown in Table 1, the ICCs for daily suicidal ideation, daily self-disgust, daily perceived stress, and daily social connectedness were 0.617, 0.634, 0.644, and 0.670, respectively. These results indicate that approximately 33.0% to 38.3% of the variance was attributable to within-person day-to-day fluctuations, whereas approximately 61.7% to 67.0% of the variance was attributable to between-person differences. Thus, all day-level variables showed meaningful variability at both the within-person and between-person levels, supporting the use of multilevel modeling in subsequent analyses.

At the between-person level, correlation analyses showed that both trait perceived stress and daily perceived stress were significantly and positively correlated with daily suicidal ideation (r = 0.343 and 0.450, respectively) and daily self-disgust (r = 0.547 and 0.750, respectively). In contrast, both trait social connectedness and daily social connectedness were significantly and negatively correlated with daily suicidal ideation (r = −0.321 and −0.469, respectively), daily perceived stress (r = −0.547 and −0.729, respectively), and daily self-disgust (r = −0.548 and −0.827, respectively). In addition, daily self-disgust was significantly and positively correlated with daily suicidal ideation (r = 0.539). Overall, the directions of the correlations among the study variables were generally consistent with the theoretical expectations of the present study.

Multicollinearity diagnostics indicated no meaningful multicollinearity at the within-person level (VIFs = 1.31–1.98), although some moderate multicollinearity was observed at the between-person level (VIFs = 3.23–6.47). Detailed multicollinearity diagnostics are presented in Supplementary Table S2.

### 3.2. Multilevel Indirect Association Model: The Role of Self-Disgust

**Figure 1:**
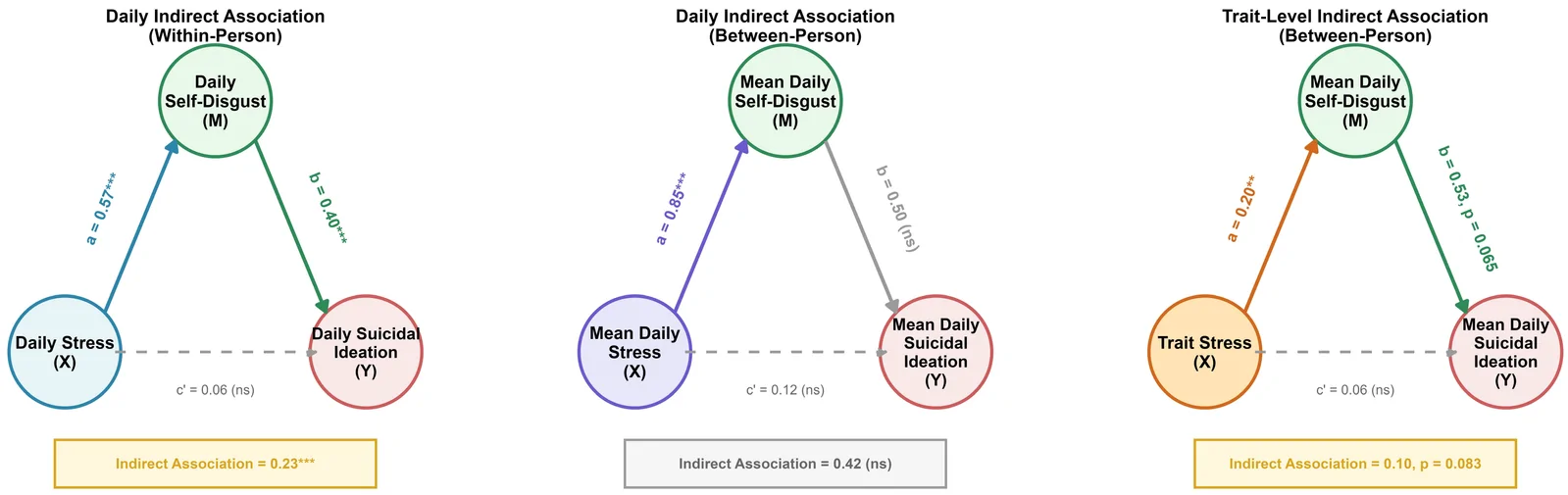
Path diagrams of the multilevel models linking perceived stress to suicidal ideation through self-disgust. Note. The left panel presents the 1-1-1 model at the within-person level, the middle panel presents the 1-1-1 model at the between-person level, and the right panel presents the 2-1-1 model. A significant within-person indirect association was observed between daily perceived stress and same-day suicidal ideation through daily self-disgust. The corresponding between-person and 2-1-1 indirect associations were not statistically significant. Path coefficients are displayed in the figure. ** *p* < 0.01, *** *p* < 0.001; ns = nonsignificant.

The results of the multilevel mediation model are presented in Table 2 and Figure 1. At the within-person level of the 1-1-1 model, daily perceived stress was significantly and positively associated with daily self-disgust, B = 0.574, SE = 0.065, *p* < 0.001, 95% CI [0.447, 0.701]. Daily self-disgust was further significantly and positively associated with same-day suicidal ideation, B = 0.403, SE = 0.089, *p* < 0.001, 95% CI [0.229, 0.577]. When daily self-disgust was included in the model, the direct association between daily perceived stress and same-day suicidal ideation was not significant, B = 0.057, SE = 0.091, *p* = 0.533, 95% CI [−0.121, 0.235]. The within-person indirect association through daily self-disgust was significant, B = 0.232, SE = 0.056, *p* < 0.001, 95% CI [0.123, 0.341].

At the between-person level of the 1-1-1 model, mean daily perceived stress was significantly and positively associated with mean daily self-disgust, B = 0.849, SE = 0.083, *p* < 0.001. Mean daily self-disgust was not significantly associated with mean daily suicidal ideation, B = 0.498, SE = 0.303, *p* = 0.101. The between-person indirect association was also not significant, B = 0.423, SE = 0.274, *p* = 0.123, 95% CI [−0.115, 0.960].

In the 2-1-1 model, trait perceived stress was significantly and positively associated with mean daily self-disgust, B = 0.196, SE = 0.061, *p* < 0.01. However, the trait-level 2-1-1 indirect association did not reach statistical significance, B = 0.103, SE = 0.059, *p* = 0.083, 95% CI [−0.013, 0.219]. Overall, the indirect association through self-disgust was significant only at the within-person level, whereas the corresponding between-person and trait-level 2-1-1 indirect associations were not statistically significant. Given that the daily variables were assessed concurrently in the same evening survey, the within-person finding should be interpreted as a same-day indirect association rather than evidence of temporal or causal mediation.

### 3.3. Multilevel Serial Indirect Association Model: The Roles of Social Connectedness and Self-Disgust

**Figure 2:**
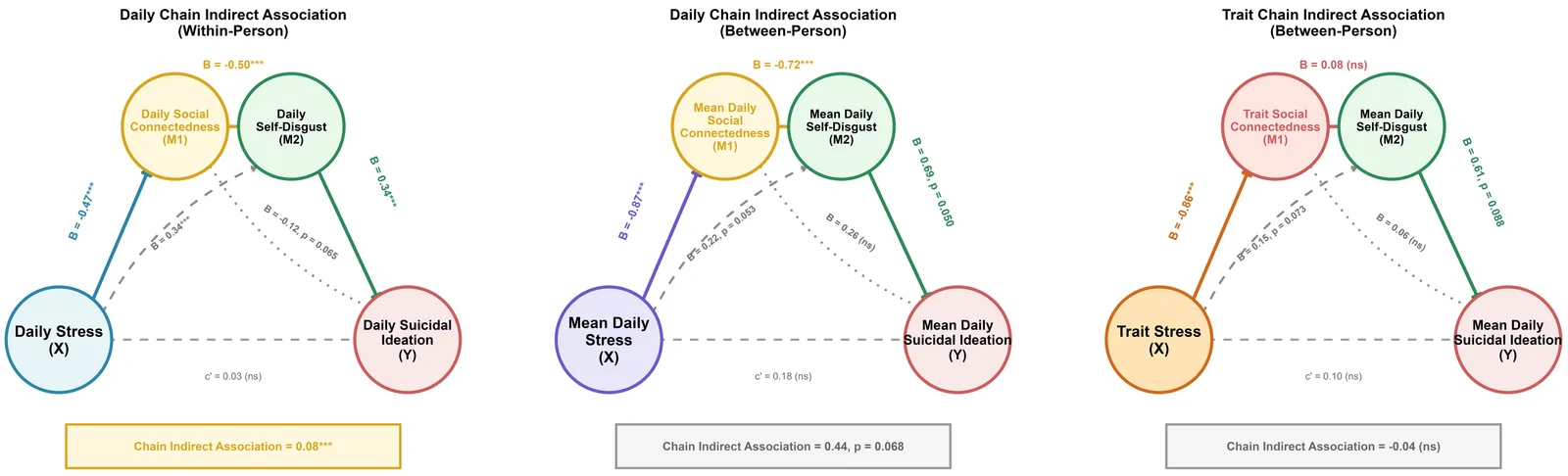
Path diagrams of the multilevel serial indirect association models linking perceived stress to suicidal ideation through social connectedness and self-disgust. Note. The left panel presents the 1-1-1 model at the within-person level, the middle panel presents the 1-1-1 model at the between-person level, and the right panel presents the 2-1-1 model. Solid arrows indicate the focal serial indirect association paths, whereas dashed or dotted arrows indicate additional direct paths included in the model. Path coefficients and exact *p* values are displayed in the figure. *** *p* < 0.001; ns = nonsignificant.

The results of the serial mediation model are presented in Table 3 and Figure 2.

At the within-person level of the 1-1-1 model, daily perceived stress was significantly and negatively associated with daily social connectedness, B = −0.471, SE = 0.044, *p* < 0.001, 95% CI [−0.557, −0.385]. Daily social connectedness was significantly and negatively associated with daily self-disgust, B = −0.503, SE = 0.045, *p* < 0.001, 95% CI [−0.591, −0.415]. Daily self-disgust was further significantly and positively associated with same-day suicidal ideation, B = 0.340, SE = 0.068, *p* < 0.001, 95% CI [0.207, 0.473]. The same-day serial indirect association between daily perceived stress and same-day suicidal ideation through daily social connectedness and daily self-disgust was significant, B = 0.081, SE = 0.019, β = 0.078, *p* < 0.001, 95% CI [0.043, 0.118]. In addition, the same-day indirect association through daily self-disgust remained significant, B = 0.115, SE = 0.027, β = 0.111, *p* < 0.001, 95% CI [0.062, 0.168].

At the between-person level of the 1-1-1 model, the serial indirect association of mean daily perceived stress with mean daily suicidal ideation through mean daily social connectedness and mean daily self-disgust was not statistically significant, B = 0.436, SE = 0.238, β = 0.445, *p* = 0.068, 95% CI [−0.032, 0.903]. In the 2-1-1 model, the serial indirect association of trait perceived stress with mean daily suicidal ideation through trait social connectedness and mean daily self-disgust was also not significant, B = −0.042, SE = 0.050, β = −0.064, *p* = 0.398, 95% CI [−0.140, 0.055]. Overall, the significant serial indirect association was observed at the within-person level, whereas the corresponding between-person and trait-level 2-1-1 serial indirect associations were not statistically significant. Specifically, on days when individuals reported higher perceived stress than usual, they also reported lower same-day social connectedness, higher same-day self-disgust, and higher same-day suicidal ideation. Given the concurrent assessment of the daily variables, this finding should be interpreted as a same-day serial indirect association rather than evidence of temporal or causal mediation.

### 3.4. Sensitivity Analyses

Sensitivity analyses additionally controlling for age, gender, educational level, and only-child status indicated that the key within-person findings remained significant. The indirect association through daily self-disgust remained significant, B = 0.115, SE = 0.041, *p* = 0.005, 95% CI [0.035, 0.195], as did the serial indirect association through daily social connectedness and daily self-disgust, B = 0.081, SE = 0.031, *p* = 0.010, 95% CI [0.019, 0.142]. The trait-level 2-1-1 serial indirect association remained nonsignificant. Full results are presented in Supplementary Table S3.

## 4. Discussion

The present study used a 7-day daily diary design to examine same-day associations among daily perceived stress, social connectedness, self-disgust, and suicidal ideation among college students. The results showed a significant within-person indirect association between daily perceived stress and same-day suicidal ideation through daily self-disgust. Further analyses showed a significant within-person same-day serial indirect association involving lower daily social connectedness and higher daily self-disgust. In contrast, the corresponding serial indirect associations at the between-person and trait levels were not statistically significant. Given the modest number of Level-2 units, these nonsignificant findings should be interpreted cautiously, as limited statistical power and estimation precision may also have contributed to the results. Overall, these findings primarily support a within-person same-day association pattern linking higher daily stress with lower social connectedness, higher self-disgust, and higher suicidal ideation. Because all daily variables were assessed concurrently in the same evening survey, these findings are best interpreted as same-day co-fluctuation patterns, and the temporal ordering of these associations remains to be established in future research.

First, the present study found a close association between daily perceived stress and suicidal ideation, which is consistent with prior findings showing that perceived stress is associated with suicidal ideation among college students (Xu et al., 2022; Yu & He, 2023). Perceived stress reflects individuals’ subjective appraisal of the uncontrollability and unpredictability of life events, as well as the insufficiency of their coping resources (Cohen et al., 1983; Lazarus & Folkman, 1984), and may therefore be more closely related to their immediate experience of psychological distress than objective stressful events. From the perspectives of the three-step theory and the integrated motivational–volitional model, stress-related pain, entrapment, and helplessness are theoretically relevant to the emergence of suicidal ideation (Klonsky et al., 2021; O’Connor & Kirtley, 2018). The present findings further suggest that this association is evident not only in between-person differences but also in day-to-day fluctuations within the same individual. In other words, on days when college students experienced higher-than-usual levels of perceived stress, they also reported higher levels of same-day suicidal ideation.

More importantly, the present study found a significant within-person indirect association between daily perceived stress and same-day suicidal ideation through self-disgust. This finding suggests that higher daily stress may co-occur with more negative self-evaluation and self-rejection, which are in turn associated with higher same-day suicidal ideation. Self-disgust is a strong negative self-conscious emotion involving disgust, rejection, and feelings of unacceptability toward the self. Compared with general negative affect, self-disgust is more directly related to impaired self-worth and self-acceptance, and may therefore represent a more proximal psychological correlate of suicidal ideation (Gao et al., 2022). Previous studies have shown that self-disgust is significantly associated with suicidal thoughts and behaviors and has been found to statistically mediate the associations between stressful experiences, such as body image disturbance and emotional abuse, and suicide-related outcomes (Akram et al., 2022; Gong & Zhou, 2025). Building on this evidence, the present study further suggests that self-disgust is not only a relatively stable correlate of suicide-related outcomes but may also show meaningful day-level covariation with perceived stress and suicidal ideation.

This finding may help clarify the association between stress and suicidal ideation. Stressful experiences may be accompanied by more negative evaluations of individuals’ own competence and worth, including perceptions of being unable to cope or not being good enough. When stress is interpreted as evidence of self-defectiveness, individuals may experience stronger self-attack and self-rejection, which may co-occur with higher levels of suicidal ideation. Relevant theories suggest that suicidal ideation may be associated not only with external difficulties, but also with individuals’ desire to escape a painful self-state (O’Connor & Kirtley, 2018; Klonsky et al., 2021). Therefore, self-disgust may represent an important self-evaluative correlate in the same-day association between stress and suicidal ideation.

Notably, the indirect association through self-disgust was significant only at the within-person level, whereas the corresponding between-person and trait-level 2-1-1 indirect associations were not statistically significant. This pattern suggests that the observed association among stress, self-disgust, and suicidal ideation may primarily reflect short-term within-person co-fluctuation rather than stable differences between individuals. Existing EMA and daily diary studies have also shown that suicidal ideation and its related risk factors can fluctuate substantially over hours or days, and that intensive longitudinal designs are useful for capturing short-term variations and within-person associations that may be difficult to detect in traditional cross-sectional research (Kivelä et al., 2022; Czyz et al., 2023; Winstone et al., 2024). Therefore, the present study highlights that, in understanding suicide risk among college students, it is important to consider not only between-person differences but also short-term variations in relevant psychological experiences within the same individual.

Regarding social connectedness, the present study further highlighted its role in the same-day associations among daily perceived stress, self-disgust, and suicidal ideation. Specifically, on days when college students experienced higher levels of stress, they reported lower same-day social connectedness; lower social connectedness was further associated with higher self-disgust, which was also associated with higher same-day suicidal ideation. This finding is consistent with previous research on social connectedness and suicidal ideation, suggesting that social connectedness may represent not only a relatively stable protective correlate but also an important interpersonal correlate of day-level negative self-evaluation. Social connectedness reflects individuals’ experiences of belonging, closeness, and acceptance within social relationships; lower social connectedness often indicates stronger interpersonal disconnection, lower belongingness, and weaker relational security (Lee et al., 2001). Among college students, insufficient belongingness has been closely associated with higher levels of suicidal ideation, with family belongingness showing a significant negative association with suicidal ideation (Ploskonka & Servaty-Seib, 2015). Similarly, recent research has suggested that the association between social connectedness and suicidal ideation may involve interpersonal psychological factors, such as thwarted belongingness and perceived burdensomeness (Gill et al., 2023), which is also consistent with the interpersonal theory of suicide, wherein thwarted belongingness is considered a risk factor for suicidal ideation (Van Orden et al., 2010). However, the ordering examined in the present study should not be regarded as the only possible ordering among these variables. These same-day associations may be reciprocal: stronger self-disgust may coincide with lower perceived relational closeness, while suicidal ideation may also be accompanied by social withdrawal or more negative perceptions of interpersonal relationships. Previous studies have similarly reported associations of self-disgust with loneliness and poorer peer relationships, as well as an association between social withdrawal and suicidal ideation (Ypsilanti et al., 2020; Liu et al., 2023; Huang et al., 2024). Thus, social connectedness, self-disgust, and suicidal ideation may co-fluctuate within the same day, with the direction of these associations remaining uncertain. Accordingly, the serial ordering examined here should be interpreted as a same-day association pattern rather than evidence of a unique temporal sequence.

From the perspective of the same-day serial association pattern observed in this study, stressful experiences may co-occur with a weaker sense of belonging, closeness, and acceptance, as well as feelings of greater interpersonal disconnection and lower relational support. Lower relational security may also be associated with lower self-worth and stronger experiences of self-denial and self-rejection. Consistent with this interpretation, Ypsilanti et al. (2019) found that self-disgust mediated the association between loneliness and depressive symptoms, suggesting a potential link between interpersonal disconnection and psychological distress involving negative self-evaluation. Therefore, reduced social connectedness may represent an important interpersonal correlate within the same-day associations among daily stress, self-disgust, and suicidal ideation. In other words, when individuals feel disconnected from others under stressful circumstances, they may also experience stronger self-denial and self-disgust, alongside higher levels of same-day suicidal ideation.

Theoretically, this study adds within-person evidence regarding same-day associations between stress and suicidal ideation, further emphasizing that suicidal ideation is not a completely stable risk state but may change alongside daily psychological experiences (Kivelä et al., 2022; Czyz et al., 2023). By incorporating social connectedness and self-disgust into the same serial indirect association model, the study identified a same-day pattern in which higher daily stress co-occurred with lower social connectedness, higher self-disgust, and higher suicidal ideation, providing a day-level perspective on short-term variations in suicidal ideation. From a practical perspective, daily perceived stress, social connectedness, and self-disgust may serve as relevant indicators for suicide-risk assessment and monitoring among college students. University mental health services may therefore benefit from attending not only to students’ stress levels but also to changes in self-disgust and perceived social connectedness. However, the present observational findings do not establish that interventions targeting these factors would reduce suicidal ideation, and further longitudinal and intervention studies are needed.

Several limitations of this study should be noted. First, the relatively small and predominantly female sample may have limited statistical power, particularly for between-person and trait-level 2-1-1 effects, and may restrict the stability and generalizability of the findings. In addition, the exclusion of participants with incomplete diary data may have introduced selection bias. Second, although the 7-day daily diary design allowed us to capture short-term fluctuations, the once-daily concurrent assessment did not establish temporal ordering or bidirectional associations among the variables; future studies could use longer follow-up periods or EMA designs with multiple assessments per day to examine lagged associations. Third, several measures were shortened and adapted for daily assessment, and suicidal ideation was assessed using a single item, warranting further validation of these brief state measures. Current mental health problems, substance misuse, and previous suicide-related history were also not assessed as eligibility or exclusion criteria. Fourth, reliance on concurrent self-report measures may have introduced common method variance and same-source bias, and suicidal ideation may have been underreported because of stigma or confidentiality concerns. The relatively high correlations among several variables may also partly reflect construct overlap or measurement redundancy; although no meaningful multicollinearity was observed at the within-person level, moderate multicollinearity was present for some between-person predictors, and the corresponding findings should therefore be interpreted cautiously. Future research incorporating larger and more diverse samples, validated measures, clinical interviews, behavioral indicators, or multi-informant data may help address these limitations.

## 5. Conclusions

Using a 7-day daily diary design, the present study identified significant same-day within-person associations among daily perceived stress, social connectedness, self-disgust, and suicidal ideation in college students. Specifically, significant within-person indirect associations through self-disgust and through the serial association involving social connectedness and self-disgust were observed. In contrast, the corresponding between-person and trait-level 2-1-1 indirect associations were generally weaker or not statistically significant, suggesting that these findings primarily reflect short-term within-person co-fluctuation. Given the concurrent assessment of the daily variables, the findings should be considered preliminary, and their temporal ordering remains to be established using larger samples, more intensive temporal designs, lagged models, and validated state-level measures.

## Figures and Tables

**Table 1:** Descriptive statistics, variance components, intraclass correlation coefficients, and correlations among the study variables.

Variable	M ± SD	Observed Range	Within-Person Variance	Between-Person Variance	ICC	1	2	3	4	5
1. Trait perceived stress	31.246 ± 8.467	12–47	—	—	—	1				
2. Trait social connectedness	73.672 ± 22.947	20–111	—	—	—	−0.856 ***	1			
3. Daily perceived stress	10.855 ± 4.106	4–20	6.058	10.953	0.644	0.587 ***	−0.547 ***	1		
4. Daily social connectedness	16.773 ± 5.183	4–24	8.950	18.172	0.670	−0.523 ***	0.609 ***	−0.729 ***	1	
5. Daily self-disgust	9.616 ± 4.886	3–21	8.810	15.276	0.634	0.547 ***	−0.548 ***	0.750 ***	−0.827 ***	1
6. Daily suicidal ideation	1.466 ± 0.853	1–5	0.281	0.454	0.617	0.343 ***	−0.321 ***	0.450 ***	−0.469 ***	0.539 ***

Note. M = mean; SD = standard deviation. Trait-level variables were measured at baseline; daily variables were measured repeatedly across the 7-day diary period. Within-person and between-person variances are reported only for the daily variables. ICC = intraclass correlation coefficient. Correlations were calculated at the between-person level using baseline trait scores and participants’ mean daily scores across the seven-day diary period (N = 61). *** *p* < 0.001.

**Table 2:** Multilevel direct and indirect associations of perceived stress with suicidal ideation through self-disgust.

Model	Level	Parameter	B	SE	*p*	95% CI	β
1-1-1	Within-person	a: Daily perceived stress → Daily self-disgust	0.574	0.065	<0.001	[0.447, 0.701]	0.567
		b: Daily self-disgust → Same-day suicidal ideation	0.403	0.089	<0.001	[0.229, 0.577]	0.394
		c′: Daily perceived stress → Same-day suicidal ideation	0.057	0.091	0.533	[−0.121, 0.235]	0.055
		Indirect association: Daily perceived stress → Daily self-disgust → Same-day suicidal ideation	0.232	0.056	<0.001	[0.123, 0.341]	0.223
		Total association: Daily perceived stress → Same-day suicidal ideation	0.288	0.082	<0.001	[0.128, 0.448]	0.278
1-1-1	Between-person	a: Mean daily perceived stress → Mean daily self-disgust	0.849	0.083	<0.001	[0.687, 1.011]	0.856
		b: Mean daily self-disgust → Mean daily suicidal ideation	0.498	0.303	0.101	[−0.097, 1.092]	0.505
		c′: Mean daily perceived stress → Mean daily suicidal ideation	0.119	0.309	0.701	[−0.487, 0.725]	0.121
		Indirect association: Mean daily perceived stress → Mean daily self-disgust → Mean daily suicidal ideation	0.423	0.274	0.123	[−0.115, 0.960]	0.432
		Total association: Mean daily perceived stress → Mean daily suicidal ideation	0.541	0.140	<0.001	[0.268, 0.815]	0.553
2-1-1		a: Trait perceived stress → Mean daily self-disgust	0.196	0.061	<0.01	[0.075, 0.316]	0.428
		b: Mean daily self-disgust → Mean daily suicidal ideation	0.526	0.285	0.065	[−0.032, 1.084]	0.354
		c′: Trait perceived stress → Mean daily suicidal ideation	0.059	0.095	0.537	[−0.128, 0.246]	0.087
		Indirect association: Trait perceived stress → Mean daily self-disgust → Mean daily suicidal ideation	0.103	0.059	0.083	[−0.013, 0.219]	0.152
		Total association: Trait perceived stress → Mean daily suicidal ideation	0.162	0.092	0.078	[−0.018, 0.342]	0.238

Note. B represents the unstandardized coefficient; SE = standard error; CI = confidence interval; β = standardized coefficient. The 1-1-1 model estimated direct and indirect associations among daily perceived stress, daily self-disgust, and same-day suicidal ideation at both the within-person and between-person levels. The 2-1-1 model examined the indirect association between trait perceived stress and mean daily suicidal ideation through mean daily self-disgust. Within-person associations reflect day-to-day deviations from an individual’s usual level, whereas between-person associations reflect differences between individuals in their average levels across the diary period.

**Table 3:** Multilevel serial indirect associations of social connectedness and self-disgust in the association between perceived stress and suicidal ideation.

Model	Level	Parameter	B	SE	*p*	95% CI	β
1-1-1	Within-person	Indirect association 1: Daily perceived stress → Daily social connectedness → Same-day suicidal ideation	0.058	0.032	0.069	[−0.005, 0.121]	0.056
		Indirect association 2: Daily perceived stress → Daily self-disgust → Same-day suicidal ideation	0.115	0.027	<0.001	[0.062, 0.168]	0.111
		Serial indirect association: Daily perceived stress → Daily social connectedness → Daily self-disgust → Same-day suicidal ideation	0.081	0.019	<0.001	[0.043, 0.118]	0.078
1-1-1	Between-person	Indirect association 1: Mean daily perceived stress → Mean daily social connectedness → Mean daily suicidal ideation	−0.227	0.305	0.458	[−0.825, 0.372]	−0.232
		Indirect association 2: Mean daily perceived stress → Mean daily self-disgust → Mean daily suicidal ideation	0.153	0.109	0.160	[−0.060, 0.366]	0.156
		Serial indirect association: Mean daily perceived stress → Mean daily social connectedness → Mean daily self-disgust → Mean daily suicidal ideation	0.436	0.238	0.068	[−0.032, 0.903]	0.445
2-1-1		Indirect association 1: Trait perceived stress → Trait social connectedness → Mean daily suicidal ideation	−0.049	0.145	0.735	[−0.332, 0.234]	−0.075
		Indirect association 2: Trait perceived stress → Mean daily self-disgust → Mean daily suicidal ideation	0.089	0.072	0.218	[−0.053, 0.231]	0.136
		Serial indirect association: Trait perceived stress → Trait social connectedness → Mean daily self-disgust → Mean daily suicidal ideation	−0.042	0.050	0.398	[−0.140, 0.055]	−0.064

Note. The table presents specific indirect associations from the serial models. The 1-1-1 model estimated indirect associations using daily measures at both the within-person and between-person levels. Within-person associations reflect same-day indirect associations among day-level deviations, whereas between-person associations reflect indirect associations among individuals’ mean daily levels across the diary period. The 2-1-1 model examined indirect associations between trait perceived stress and mean daily suicidal ideation through trait social connectedness and mean daily self-disgust. Only specific indirect associations are presented in this table; full path coefficients are provided in the Supplementary Materials. B represents the unstandardized coefficient; SE = standard error; CI = confidence interval; β = standardized coefficient.

## Data Availability

The data presented in this study are not publicly available due to privacy and ethical restrictions. The data contain sensitive participant information and were collected under conditions of confidentiality; therefore, they cannot be shared publicly.

## Supplementary Materials

The following supporting information can be downloaded at: https://www.mdpi.com/article/10.3390/bs16081284/s1, Table S1: Exact item wording, response options, scoring directions, and possible score ranges of the daily measures; Table S2: Multicollinearity diagnostics for the within-person, between-person, and trait-level models; Table S3: Sensitivity analyses of the key indirect associations after adjustment for demographic covariates; Table S4: Results of the multilevel indirect association models including residual variances; Table S5: Results of the multilevel serial indirect association models.
