# Supplementary material for: Same-Day Associations Between Perceived Stress and Suicidal Ideation Among College Students: The Roles of Social Connectedness and Self-Disgust in a 7-Day Diary Study
Source: Behav Sci (Basel). 2026 Jul 28;16(8):1284. doi: 10.3390/bs16081284 (PMC13509533; doi:10.3390/bs16081284)
Supplement: Supplementary file 1 [file behavsci-16-01284-s001.zip › behavsci-4419440-supplementary.pdf]

Supplementary Table S1. Exact Item Wording, Response Options, Scoring Directions, and Possible Score Ranges of the Daily Measures.

Measure	Exact item wording	Response options	Scoring direction	Possible score range
Daily suicidal ideation	Today, how many times have you considered ending your life?	1 = Never; 2 = Rarely (once); 3 = Sometimes (twice); 4 = Often (three to four times); 5 = Very often (five or more times)	The item was scored directly from 1 to 5, with higher scores indicating more frequent same-day suicidal ideation. No reverse scoring was applied.	1–5
Daily self-disgust	1. Today, I hated being myself.<br>2. Today, I liked the way I looked.<br>3. Today, I felt that people did not like me.	1 = Strongly agree; 2 = Agree; 3 = Somewhat agree; 4 = Neutral; 5 = Somewhat disagree; 6 = Disagree; 7 = Strongly disagree	Given the direction of the response scale, Items 1 and 3 were reverse-coded (1 ↔ 7, 2 ↔ 6, 3 ↔ 5, 4 unchanged), whereas Item 2 was scored as reported. The three item scores were summed, with higher scores indicating higher levels of same-day self-disgust.	3–21
Daily social connectedness	1. Today, I felt close to people around me.<br>2. Today, when I was with my peers, I felt like an outsider.<br>3. Today, I felt understood by people I knew.<br>4. Today, I felt distant from other people.	1 = Strongly disagree; 2 = Moderately disagree; 3 = Slightly disagree; 4 = Slightly agree; 5 = Moderately agree; 6 = Strongly agree	Items 2 and 4 were reverse-coded (1 ↔ 6, 2 ↔ 5, 3 ↔ 4). The four item scores were summed, with higher scores indicating higher levels of same-day social connectedness.	4–24
Daily perceived stress	1. Today, I was upset because of something that happened unexpectedly.<br>2. Today, I was able to successfully handle irritating things in life.<br>3. Today, I felt that I was on top of things.<br>4. Today, I felt that difficulties were piling up so high that I could not overcome them.	1 = Never; 2 = Occasionally; 3 = Sometimes; 4 = Often; 5 = Always	Items 2 and 3 were reverse-coded (1 ↔ 5, 2 ↔ 4, 3 unchanged). The four item scores were summed, with higher scores indicating higher levels of same-day perceived stress.	4–20

Note. All daily items were administered in Chinese. English translations are presented here for reporting purposes. Item wording was adapted to refer specifically to participants' experiences "today." Reverse-coded items were recoded before total scores were calculated. Higher total scores consistently indicate higher levels of the corresponding construct.

Supplementary Table S2. Multicollinearity diagnostics for the within-person, between-person, and trait-level models.

Model equation	Predictor	VIF (95% CI)	Tolerance (95% CI)
**Within-person: self-disgust equation**	Daily perceived stress (within-person deviation)	1.315 [1.194, 1.511]	0.761 [0.662, 0.838]
	Daily social connectedness (within-person deviation)	1.315 [1.194, 1.511]	0.761 [0.662, 0.838]
**Within-person: suicidal ideation equation**	Daily perceived stress (within-person deviation)	1.534 [1.372, 1.767]	0.652 [0.566, 0.729]
	Daily social connectedness (within-person deviation)	1.767 [1.563, 2.045]	0.566 [0.489, 0.640]
	Daily self-disgust (within-person deviation)	1.980 [1.739, 2.299]	0.505 [0.435, 0.575]
**Between-person: self-disgust equation**	Mean daily perceived stress	3.229 [2.272, 4.904]	0.310 [0.204, 0.440]
	Mean daily social connectedness	3.229 [2.272, 4.904]	0.310 [0.204, 0.440]
**Between-person: suicidal ideation equation**	Mean daily perceived stress	3.646 [2.550, 5.518]	0.274 [0.181, 0.392]
	Mean daily social connectedness	6.378 [4.289, 9.793]	0.157 [0.102, 0.233]
	Mean daily self-disgust	6.473 [4.350, 9.942]	0.154 [0.101, 0.230]
**2-1-1 trait-level: self-disgust equation**	Trait perceived stress	3.751 [2.601, 5.726]	0.267 [0.175, 0.384]
	Trait social connectedness	3.751 [2.601, 5.726]	0.267 [0.175, 0.384]
**2-1-1 trait-level: suicidal ideation equation**	Trait perceived stress	4.032 [2.796, 6.121]	0.248 [0.163, 0.358]
	Trait social connectedness	4.271 [2.947, 6.495]	0.234 [0.154, 0.339]
	Mean daily self-disgust	2.458 [1.797, 3.667]	0.407 [0.273, 0.557]

Note. VIF = variance inflation factor; CI = confidence interval. Within-person predictors represent day-level deviations from each participant's own mean, whereas between-person predictors represent participants' mean daily levels across the diary period. The 2-1-1 models include trait-level predictors at Level 2. VIF values ranged from 1.31 to 1.98 in the within-person equations, from 3.23 to 6.47 in the between-person equations, and from 2.46 to 4.27 in the 2-1-1 trait-level equations.

Supplementary Table S3. Sensitivity analyses of the key indirect associations after adjustment for demographic covariates.

Model	Level	Indirect association	B	SE	p	95% CI	β
1-1-1	Within-person	Daily perceived stress → Daily self-disgust → Same-day suicidal ideation	0.115	0.041	0.005	[0.035, 0.195]	0.111
		Daily perceived stress → Daily social connectedness → Daily self-disgust → Same-day suicidal ideation	0.081	0.031	0.010	[0.019, 0.142]	0.078
2-1-1	Trait-level	Trait perceived stress → Trait social connectedness → Mean daily self-disgust → Mean daily suicidal ideation	-0.043	0.056	0.448	[-0.153, 0.068]	-0.065

Note. Sensitivity analyses simultaneously included age, gender, educational level, and only-child status as Level-2 covariates predicting social connectedness, self-disgust, and suicidal ideation. Age was grand-mean centered, whereas gender, educational level, and only-child status were dummy coded. B = unstandardized coefficient; SE = standard error; CI = confidence interval; β = standardized coefficient.

Table S4. Results of the multilevel indirect association models including residual variances.

Model	Level	Parameter	Estimate	SE	*p*	95% CI LL	95% CI UL	Std. Est.
1-1-1	Within-person	a: Daily stress → Daily self-disgust	0.574	0.065	< .001	0.447	0.701	0.567
		b: Daily self-disgust → Daily suicidal ideation	0.403	0.089	< .001	0.229	0.577	0.394
		c′: Daily stress → Daily suicidal ideation	0.057	0.091	.533	-0.121	0.235	0.055
		Indirect association: Daily stress → Daily self-disgust → Daily suicidal ideation	0.232	0.056	< .001	0.123	0.341	0.223
		Total association: Daily stress → Daily suicidal ideation	0.288	0.082	< .001	0.128	0.448	0.278
		Residual variance of daily self-disgust	0.251	0.040	< .001	0.171	0.330	0.679
		Residual variance of daily suicidal ideation	0.315	0.071	< .001	0.176	0.455	0.817
	Between-person	a: Mean daily stress → Mean daily self-disgust	0.849	0.083	< .001	0.687	1.011	0.856
		b: Mean daily self-disgust → Mean daily suicidal ideation	0.498	0.303	.101	-0.097	1.092	0.505
		c′: Mean daily stress → Mean daily suicidal ideation	0.119	0.309	.701	-0.487	0.725	0.121
		Indirect association: Mean daily stress → Mean daily self-	0.423	0.274	.123	-0.115	0.960	0.432

Model	Level	Parameter	Estimate	SE	*p*	95% CI LL	95% CI UL	Std. Est.
		disgust → Mean daily suicidal ideation						
		Total association: Mean daily stress → Mean daily suicidal ideation	0.541	0.140	< .001	0.268	0.815	0.553
		Residual variance of mean daily self-disgust	0.169	0.046	< .001	0.079	0.258	0.268
		Residual variance of mean daily suicidal ideation	0.383	0.093	< .001	0.200	0.566	0.626
2-1-1	Within-person	a: Daily stress → Daily self-disgust	0.598	0.058	< .001	0.484	0.713	0.766
		b: Daily self-disgust → Daily suicidal ideation	0.404	0.089	< .001	0.229	0.578	0.469
		c′: Daily stress → Daily suicidal ideation	0.067	0.090	.455	-0.109	0.242	0.099
		Residual variance of daily self-disgust	0.251	0.040	< .001	0.172	0.330	0.412
		Residual variance of daily suicidal ideation	0.315	0.071	< .001	0.176	0.455	0.699
	Between-person	a: Trait stress → Mean daily self-disgust	0.196	0.061	< .01	0.075	0.316	0.428
		b: Mean daily self-disgust → Mean daily suicidal ideation	0.526	0.285	.065	-0.032	1.084	0.354
		c′: Trait stress → Mean daily suicidal ideation	0.059	0.095	.537	-0.128	0.246	0.087
		Indirect association: Trait stress → Mean daily self-disgust → Mean daily suicidal ideation	0.103	0.059	.083	-0.013	0.219	0.152
		Total association: Trait stress → Mean daily suicidal ideation	0.162	0.092	.078	-0.018	0.342	0.238
		Residual variance of mean daily self-disgust	0.170	0.040	< .001	0.092	0.248	0.817
		Residual variance of mean daily suicidal ideation	0.386	0.097	< .001	0.197	0.576	0.841

Note. The 1-1-1 model tested the multilevel mediation among daily stress, daily self-disgust, and daily suicidal ideation at both the within- and between-person levels. The 2-1-1 model tested the cross-level mediation among trait stress, daily self-disgust, and daily suicidal ideation. SE = standard error; CI = confidence interval; LL = lower limit; UL = upper limit; Std. Est. = standardized estimate. Residual variances refer to within- and between-person residual variances. In the 2-1-1 model, the within-person component was included only as a control path; therefore, within-person indirect and total associations were not reported.

Table S5. Results of the multilevel serial indirect association models.

Model	Level	Parameter	Estimate	SE	*p*	95% CI LL	95% CI UL	Std. Est.
1-1-1	Within-person	a1: Daily stress → Daily social connectedness	-0.471	0.044	< .001	-0.557	-0.385	-0.489
		d1: Daily social connectedness → Daily self-disgust	-0.503	0.045	< .001	-0.591	-0.415	-0.478
		a2: Daily stress → Daily self-disgust	0.337	0.043	< .001	0.253	0.422	0.333
		b1: Daily self-disgust → Daily suicidal ideation	0.340	0.068	< .001	0.207	0.473	0.333
		d2: Daily social connectedness → Daily suicidal ideation	-0.124	0.067	.065	-0.256	0.008	-0.115
		c′: Daily stress → Daily suicidal ideation	0.034	0.060	.568	-0.084	0.153	0.033
		Indirect association 1: Daily stress → Daily social connectedness → Daily suicidal ideation	0.058	0.032	.069	-0.005	0.121	0.056
		Indirect association 2: Daily stress → Daily self-disgust → Daily suicidal ideation	0.115	0.027	< .001	0.062	0.168	0.111
		Serial indirect association: Daily stress → Daily social connectedness → Daily self-disgust → Daily suicidal ideation	0.081	0.019	< .001	0.043	0.118	0.078
		Total indirect association	0.254	0.040	< .001	0.175	0.332	0.245
		Total association	0.288	0.052	< .001	0.186	0.390	0.278
	Between-person	a1: Mean daily stress → Mean daily social connectedness	-0.874	0.079	< .001	-1.028	-0.720	-0.857
		d1: Mean daily social connectedness → Mean daily self-disgust	-0.719	0.111	< .001	-0.937	-0.501	-0.739
		a2: Mean daily stress → Mean daily self-disgust	0.220	0.114	.053	-0.003	0.444	0.222
		b1: Mean daily self-disgust → Mean daily suicidal ideation	0.693	0.354	.050	-0.001	1.387	0.703
		d2: Mean daily social connectedness → Mean daily suicidal ideation	0.259	0.348	.456	-0.423	0.942	0.270
		c′: Mean daily stress → Mean daily suicidal ideation	0.179	0.238	.452	-0.288	0.647	0.183
		Indirect association 1: Mean daily stress → Mean daily social connectedness → Mean daily suicidal ideation	-0.227	0.305	.458	-0.825	0.372	-0.232
		Indirect association 2: Mean daily stress → Mean daily self-	0.153	0.109	.160	-0.060	0.366	0.156

Model	Level	Parameter	Estimate	SE	*p*	95% CI LL	95% CI UL	Std. Est.
		disgust → Mean daily suicidal ideation						
		Serial indirect association: Mean daily stress → Mean daily social connectedness → Mean daily self-disgust → Mean daily suicidal ideation	0.436	0.238	.068	-0.032	0.903	0.445
		Total indirect association	0.362	0.210	.085	-0.050	0.774	0.370
		Total association	0.541	0.115	< .001	0.315	0.767	0.553
2-1-1	Between-person	a1: Trait stress → Trait social connectedness	-0.856	0.066	< .001	-0.986	-0.727	-0.856
		d1: Trait social connectedness → Mean daily self-disgust	0.081	0.083	.328	-0.081	0.244	0.278
		a2: Trait stress → Mean daily self-disgust	0.147	0.082	.073	-0.013	0.307	0.504
		b1: Mean daily self-disgust → Mean daily suicidal ideation	0.605	0.355	.088	-0.090	1.300	0.270
		d2: Trait social connectedness → Mean daily suicidal ideation	0.057	0.169	.735	-0.274	0.388	0.088
		c′: Trait stress → Mean daily suicidal ideation	0.095	0.173	.583	-0.244	0.435	0.146
		Indirect association 1: Trait stress → Trait social connectedness → Mean daily suicidal ideation	-0.049	0.145	.735	-0.332	0.234	-0.075
		Indirect association 2: Trait stress → Mean daily self-disgust → Mean daily suicidal ideation	0.089	0.072	.218	-0.053	0.231	0.136
		Serial indirect association: Trait stress → Trait social connectedness → Mean daily self-disgust → Mean daily suicidal ideation	-0.042	0.050	.398	-0.140	0.055	-0.064
		Total indirect association	-0.002	0.155	.989	-0.305	0.301	-0.003
		Total association	0.093	0.093	.315	-0.089	0.275	0.143

Note. The 1-1-1 model tested the serial mediation among daily perceived stress, daily social connectedness, daily self-disgust, and daily suicidal ideation at both the within- and between-person levels. The 2-1-1 model tested the serial mediation among trait perceived stress, trait social connectedness, mean daily self-disgust, and mean daily suicidal ideation. SE = standard error; CI = confidence interval; LL = lower limit; UL = upper limit; Std. Est. = standardized estimate.
